# Linking remote sensing parameters to CO_2_ assimilation rates at a leaf scale

**DOI:** 10.1007/s10265-021-01313-4

**Published:** 2021-05-21

**Authors:** Kouki Hikosaka, Katsuto Tsujimoto

**Affiliations:** Graduate School of Life Sciences, Tohoku University, Aoba, Sendai, 980-8578 Japan

**Keywords:** Chlorophyll fluorescence, Gas exchange, Light energy partitioning, Non-photochemical quenching, Photochemical reflectance index (PRI)

## Abstract

Solar-induced chlorophyll fluorescence (SIF) and photochemical reflectance index (PRI) are expected to be useful for remote sensing of photosynthetic activity at various spatial scales. This review discusses how chlorophyll fluorescence and PRI are related to the CO_2_ assimilation rate at a leaf scale. Light energy absorbed by photosystem II chlorophylls is allocated to photochemistry, fluorescence, and heat dissipation evaluated as non-photochemical quenching (NPQ). PRI is correlated with NPQ because it reflects the composition of xanthophylls, which are involved in heat dissipation. Assuming that NPQ is uniquely related to the photochemical efficiency (quantum yield of photochemistry), photochemical efficiencies can be assessed from either chlorophyll fluorescence or PRI. However, this assumption may not be held under some conditions such as low temperatures and photoinhibitory environments. Even in such cases, photosynthesis may be estimated more accurately if both chlorophyll fluorescence and PRI are determined simultaneously. To convert from photochemical efficiency to CO_2_ assimilation, environmental responses in stomatal conductance also need to be considered. Models linking chlorophyll fluorescence and PRI with CO_2_ assimilation rates will contribute to understanding and future prediction of the global carbon cycle.

## Introduction

Carbon assimilation by photosynthetic organisms, i.e., gross primary production (GPP), is one of the most important drivers of global carbon cycling and climate. Accurate estimation of GPP is indispensable for understanding and future projection of global climate. Thus far, a number of studies have estimated global GPP, but the presented values have large variation among the studies (Baldochi et al. 2015), probably due to lack of an accurate method. At an ecosystem scale, GPP can be accurately estimated by biometric method or eddy covariance. However, because such methods are applicable only to local ecosystems, many observation points and assumptions are required to evaluate GPP at regional or global scales.

Satellite observation is a unique method to evaluate ecosystem functions at a global scale as it can directly observe global vegetation (Schimel et al. 2015). Various indices have been used to evaluate ecosystem functions. For example, the normalized difference vegetation index (NDVI; Rouse et al. 1974), which reflects absorption spectra of chlorophylls (Chl), has been used for estimation of GPP (Field et al. 1995). However, most of such indices detect the amount of photosynthetic pigment in the vegetation only. Even when the Chl content is the same, leaf CO_2_ assimilation rates change depending on the environmental variables at the site. Furthermore, photosynthetic traits have a large variation among species and even within a species depending on growth conditions. Such variations cannot necessarily be detected with vegetation indices.

Recently, solar-induced Chl fluorescence (SIF) and photochemical reflectance index (PRI) have attracted many remote sensing researchers. Both of them are considered to change depending on the status of the photosynthetic apparatus and can be obtained remotely with a spectroradiometer. In particular, SIF has been used to predict GPP with empirical relationships (Li and Xiao 2019) or theoretical radiative transfer models such as Soil-Canopy-Observation of Photosynthesis and Energy fluxes model (SCOPE; van der Tol et al. 2009a, b, 2014). In this review, we discuss how these variables are related to photosynthesis and the environment. This article comprises of five sections. In the first and second sections, basics of light energy partitioning in photosystem II (PSII) and gas exchange are discussed, respectively, both of which have frequently been explained in previous review articles and textbooks (e.g., Baker 2008; Hikosaka et al. 2016; Ogawa and Sonoike 2021; Porcar-Castell et al. 2014; Ruban 2017; von Caemmerer 2000). Therefore, readers who have studied these issues may skip these sections. The third and fourth sections introduce remote sensing parameters and their environmental responses, respectively. In the fifth section, we discuss theoretical linkages between the remote sensing parameters and leaf gas exchange rates.

## Light energy partitioning

The first step of photosynthesis is the absorption of light energy by Chl. Excitation energy is transferred from the antenna Chl molecule to the reaction center of the photosystem. An electron is released from the excited reaction center to electron acceptors and finally NADP^+^ is reduced. Through the electron transport, protons are accumulated in the thylakoid lumen, which are utilized for ATP synthesis. NADPH and ATP are utilized for CO_2_ fixation in the Calvin-Benson cycle and photorespiration.

**Fig. 1:**
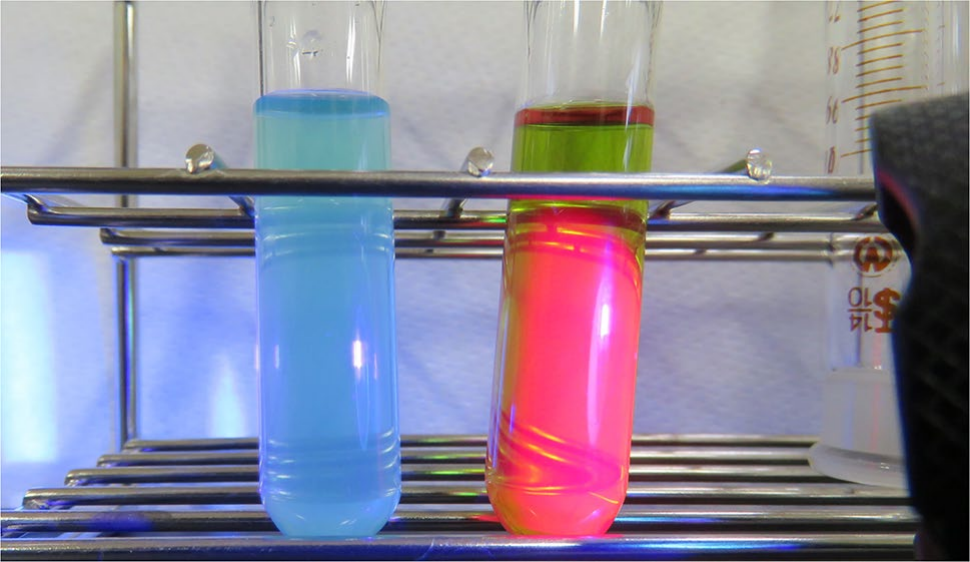
Photograph of extracted chlorophyll (right) and a blue-green pigment viridian (Cr_2_O(OH)_4_) (left) illuminated by ultraviolet light. Fluorescence induced by the ultraviolet light is seen as red light from chlorophyll but not from viridian. Chlorophyll was extracted with dimethylformamide and viridian was solubilized in water

In PSII, most of the absorbed energy is utilized for photochemical reaction in the reaction center and eventually for CO_2_ fixation and photorespiration under non-stressful conditions. However, the absorbed energy is also allocated to other processes such as fluorescence and heat loss (Fig. 1). Under stressful conditions, where most of the absorbed energy cannot be consumed by CO_2_ fixation nor photorespiration, plants allocate the absorbed energy to heat dissipation systems so as to eliminate the excess energy safely.

Energy allocation to different processes in PSII can be expressed using the rate constants (*K*) for the processes (Baker 2008). Quantum yield of the process *i* (Φ_*i*_, energy consumed by the process *i* per the total absorbed energy by PSII Chl) is expressed as follows:

1 $${\Phi }_{i} = \frac{{K_{i} }}{{K_{{\text{P}}} + K_{{\text{F}}} + K_{{\text{D}}} + K_{{\text{N}}} }}$$

where *K*_*i*_ is the rate constant of energy consumption of the process *i*, which is either of PSII photochemical reaction (P), Chl fluorescence (F), variable heat dissipation (N), or constitutive energy loss (D). The constitutive energy loss may include the energy lost as heat, conversion of Chl into the triplet form, and so on (Porcar-Castell et al. 2014). Variable heat dissipation increases when the excess energy that is not utilized in CO_2_ fixation nor photorespiration is increased and acts as a protective mechanism for PSII from the excess light energy (Demmig-Adams and Adams 1996).

**Fig. 2:**
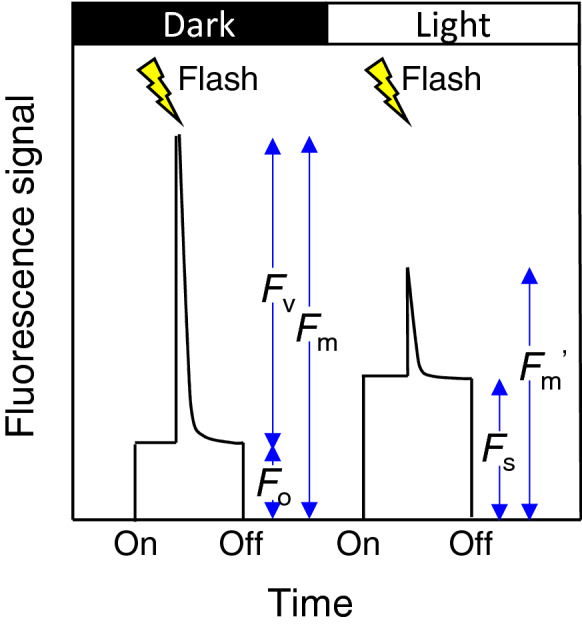
Typical trace of chlorophyll fluorescence in the dark (left) and in the light (right). Fluorescence is detected after turning on of measuring beam (‘On’) and lost after turning off (‘Off’). Flash is a strong light that saturates electron acceptors in photosystem II. Fluorescence signal is proportional to the fluorescence yield (Φ_F_) but the coefficient varies depending on various factors such as strength of measuring beam and distance between the leaf and probe

Chl fluorometers with the pulse amplitude modulation (PAM) system have enabled us to evaluate energy allocation to these processes (Schreiber et al. 1995). The PAM system provides modulated light (measuring beam) and detects the fluorescence that is induced by the measuring beam only. Because the fluorescence induced by the measuring beam is also modulated, the PAM system can distinguish it from reflection and fluorescence induced by other light. If the intensity of the measuring beam is constant, the fluorescence level detected by the PAM system is always proportional to the quantum yield of Chl fluorescence (Φ_F_; energy emitted as fluorescence per unit absorbed energy). The PAM system also provides saturating flashes (Fig. 2), which is strong enough to ‘close’ (reduce) all of the electron acceptors (Q_A_) of PSII. Therefore, photochemistry does not occur and *K*_P_ is zero during the flash. The fluorescence level increases during the flash because the absorbed energy is not utilized by photochemistry and allocated to other processes including fluorescence (Fig. 2). The quantum yield of PSII photochemistry can be obtained from the fluorescence levels with and without the flash as (Genty et al. 1989; Kitajima and Butler 1975):

2 $$\frac{{F_{{\text{v}}} }}{{F_{{\text{m}}} }} = \frac{{F_{{\text{m}}} - F_{{\text{o}}} }}{{F_{{\text{m}}} }}$$

and

3 $${\Phi }_{{\text{P}}} = \frac{{F_{{\text{m}}}^{^{\prime}} - F_{{\text{s}}} }}{{F_{{\text{m}}}^{^{\prime}} }}$$

where *F*_o_ is the fluorescence level of the dark-adapted leaves in the dark, *F*_m_ is the fluorescence level of dark-adapted leaves during the flash, *F*_s_ is the steady-state fluorescence level in the light, and *F*_m_' is the fluorescence level in the light-adapted leaves during the flash (Fig. 2). *F*_v_/*F*_m_ and Φ_P_ are defined as the quantum yield of PSII photochemistry in the dark (or the maximal quantum yield; Φ_Pmax_) and in the light, respectively.

The variable heat dissipation is assessed as the non-photochemical quenching (NPQ); an increase in the energy allocation to the heat dissipation decreases the fluorescence level. NPQ is defined as follows (Bilger and Björkman 1990):

4 $${\text{NPQ}} = \frac{{F_{{\text{m}}} }}{{F_{{\text{m}}}^{^{\prime}} }} - 1$$

Based on Eq. 1, NPQ is also equal to *K*_N_/(*K*_F_ + *K*_D_) (Porcar-Castell et al. 2014). Since *K*_F_ and *K*_D_ are assumed to be constant irrespective of environmental conditions, NPQ is proportional to *K*_N_. Equations to derive the quantum yield of NPQ (Φ_NPQ_) and that of fluorescence + constitutive energy loss (Φ_NO_) are given by Kramer et al. (2004).

NPQ is known to consist of various mechanisms. The most important mechanism is known as the energy-dependent quenching (*q*_E_) (Müller et al. 2001). In stress environments such as strong light, extremely low and high temperatures and drought, the rate of energy consumption for CO_2_ fixation and photorespiration is lower than the potential rate of photochemistry and electron transport. In this situation, absorbed energy is excessive and potentially harmful for the photosynthetic apparatus. Relatively low energy consumption rate under the strong light causes acidification of lumen, leading to a protonation of PsbS, a subunit of PSII. In addition, de-epoxidation of violaxanthin is induced, and antheraxanthin and zeaxanthin are produced (the xanthophyll cycle). These protonated PsbS and de-epoxidated xanthophylls are considered to play an important role in heat dissipation. Induction and relaxation of *q*_E_ are completed within 10–20 min. *q*_E_ is considered as an important mechanism to protect plant tissues from stresses. In fact, mutants lacking genes related to protonation of PsbS or to the xanthophyll cycle are susceptible to stresses (Niyogi 1999).

Another important component of NPQ is photoinhibition. Photoinhibition of PSII has been defined as a decrease in PSII activity due to strong light, assessed with CO_2_ assimilation rate, O_2_ evolution rate, electron transport rate, or *F*_v_/*F*_m_ values. Although this definition includes energy-dependent NPQ in a broad sense (e.g., ‘dynamic photoinhibition’ defined by Osmond 1994), photoinhibition is generally used for the irreversible inhibition that does not recover without chloroplast protein synthesis (Tyystjärvi 2013). PSII is susceptible to strong light and the rate constant of photodamage is very high. For example, if the recovery of damaged PSII is artificially inhibited, more than half of the PSII loses its activity within several hours under > 1000 µmol m^−2^ s^−1^ photosynthetic photon flux density (PPFD) (Aro et al. 1993b; Kato et al. 2002). On the other hand, damaged PSII is recovered by the de novo synthesis of D1 protein and incorporation back into the thylakoid membrane after degradation of damaged D1 protein (Aro et al. 1993a). This fast turnover of damaged PSII contributes to the maintenance of active PSII at high light (Aro et al. 1993b). However, the PSII repair process is often inhibited under environmental stresses, leading to an accumulation of damaged PSII, i.e., photoinhibition (Aro et al. 1993a; Murata et al. 2007; Takahashi and Murata 2008; Tsonev and Hikosaka 2003). Photoinhibited leaves have not only lower activities of PSII but also lower CO_2_ exchange rates even at saturating PPFD, where photosynthesis is not light-limited (Hikosaka et al. 2004).

Other mechanisms are also involved in NPQ. For example, phosphorylated light harvesting Chl-protein complexes (LHCII) move and allocate the absorbed energy to photosystem I (PSI), known as the state transition. Although state transition is an important mechanism for photoprotection in algae, its contribution in land plants for photoprotection is believed to be minor (Müller et al. 2001). See Malnoë (2018) for a review of other components of NPQ.

It is known that *q*_E_ is relaxed in the dark within a relatively short time whereas PSII repair is very slow in the dark. It was suggested that three components of NPQ can be distinguished by the difference in relaxation time in the dark (Quick and Stitt 1989; Walters and Horton 1991). The components with half-relaxation time of 1 min, 5 min, and hours were considered as *q*_E_, quenching by state transition (*q*_T_), and quenching by photoinhibition (*q*_I_), respectively. Maxwell and Johnson (2000) proposed equations to divide NPQ into the fast and slow relaxation components.

## Gas exchange at a leaf scale

In C_3_ photosynthesis, the first step of CO_2_ assimilation is the carboxylation of ribulose 1,5-bisphosphate (RuBP), which is catalyzed by ribulose-1,5-bisphosphate carboxylase/oxygenase (Rubisco), where one molecule of CO_2_ is associated with RuBP and two molecules of 3-phosphoglyceric acid (PGA) are produced. Triose-phosphate (TP) is produced from PGA with consumption of ATP and NADPH. A part of TP is used for synthesis of sugars or starch and RuBP is regenerated from the remains with consumption of ATP, known as the Calvin-Benson cycle. Rubisco also catalyzes RuBP oxygenation, where one molecule of O_2_ is associated with RuBP and one molecule of PGA and 2-phosphoglycolate are produced. From two molecules of 2-phosphoglycolate, one molecule of PGA is regenerated through the glyceric acid pathway and one molecule of CO_2_ is released, known as photorespiration. The active site of carboxylation and oxygenation in Rubisco is identical, so that O_2_ and CO_2_ compete for the active site. The CO_2_ assimilation rate (A) is expressed as follows (Farquhar et al. 1980):

5 $$A = V_{c} - 0.5V_{o} - R_{d}$$

where *V*_c_ and *V*_o_ are the rate of carboxylation and oxygenation, respectively, and *R*_d_ is the respiration rate in the light, which is known to be lower than the respiration rate in the dark (Brooks and Farquhar 1985; Villar et al. 1994).

CO_2_ assimilation is potentially limited by different reactions depending on the environment. When CO_2_ concentration is low, RuBP is saturated and RuBP carboxylation is the limiting step. When light intensity is low, RuBP regeneration limits photosynthesis. Utilization of photoassimilates often limits CO_2_ assimilation rate (i.e., sink limitation). According to the model of Farquhar et al. (1980) and Sharkey (1985), CO_2_ assimilation rate is expressed by the following equations:

6 $$A_{{\text{c}}} = \frac{{V_{{{\text{cmax}}}} (C_{{\text{c}}} - {\Gamma }^{*} )}}{{C_{{\text{c}}} + K_{{\text{c}}} (1 + O/K_{{\text{o}}} )}} - R_{{\text{d}}}$$

7 $$A_{{\text{j}}} = \frac{{J(C_{{\text{c}}} - {\Gamma }^{*} )}}{{4\left( {C_{c} + 2{\Gamma }^{*} } \right)}} - R_{{\text{d}}}$$

8 $$A_{{\text{t}}} = 3P_{{\text{p}}} - R_{{\text{d}}}$$

where *A*_c_, *A*_j_, and *A*_t_ are the RuBP-saturated, RuBP-limited, and TP utilization-limited rate of CO_2_ assimilation, respectively, *V*_cmax_ is the maximum rate of RuBP carboxylation, *C*_c_ is the CO_2_ partial pressure in the carboxylation site, Γ* is the CO_2_ compensation point in the absence of the respiration in the light, *K*_c_ and *K*_o_ are the Michaelis constants for CO_2_ and O_2_, *J* is the electron transport rate to provide reducing power to the Calvin-Benson cycle and photorespiration (note that *J* should not include electron transport for other processes. See below), and *P*_p_ is the rate of TP utilization. The CO_2_ assimilation rate is given by the minimum of *A*_c_, *A*_j_, and *A*_t_.

9 $$A = {\text{min}}(A_{{\text{c}}} ,A_{{\text{j}}} ,A_{{\text{t}}} )$$

*J* changes depending on light intensity, which can be expressed as a non-rectangular hyperbola:

10 $$J = \frac{{\phi_{{\text{j}}} I + J_{{{\text{max}}}} - \sqrt {\left( {\phi_{{\text{j}}} I + J_{{{\text{max}}}} } \right)^{2} - 4\phi_{j} IJ_{{{\text{max}}}} \theta_{{\text{j}}} } }}{{2\theta_{{\text{j}}} }}$$

where *I* is photosynthetically active photon flux density (PPFD) intercepted by the leaf, *J*_max_ is the light-saturated rate of electron transport, *θ*_j_ is the convexity of the curve and *ϕ*_j_ is the initial slope. Temperature dependence of *V*_cmax_, Γ*, *K*_c_, *K*_o_, and *J*_max_ (represented as *f* in the following equations) can be expressed by the Arrhenius equation or the peak equation if the suppression at high temperatures is observed.

11 $$f = f_{{{\text{ref}}}} {\text{exp}}\left[ {\frac{{E_{{\text{a}}} \left( {T_{{\text{k}}} - T_{{{\text{ref}}}} } \right)}}{{{\varvec{R}}T_{{\text{k}}} T_{{{\text{ref}}}} }}} \right]$$

12 $$f = \frac{{f_{{{\text{ref}}}} {\text{exp}}\left[ {\frac{{E_{{\text{a}}} \left( {T_{{\text{k}}} - T_{{{\text{ref}}}} } \right)}}{{{\varvec{R}}T_{{\text{k}}} T_{{{\text{ref}}}} }}} \right]\left[ {1 + {\text{exp}}\left( {\frac{{T_{{{\text{ref}}}} {\Delta }S - H_{{\text{d}}} }}{{{\varvec{R}}T_{{{\text{ref}}}} }}} \right)} \right]}}{{1 + {\text{exp}}\left( {\frac{{T_{{\text{k}}} {\Delta }S - H_{{\text{d}}} }}{{{\varvec{R}}T_{{\text{k}}} }}} \right)}}$$

where *T*_k_ and *T*_ref_ are leaf temperature and reference temperature in Kelvin, respectively, *f* and *f*_ref_ correspond to the value of *f* at *T*_k_ = 0 and the reference temperature, respectively, *E*_a_ is the activation energy of *f*, ***R*** is the universal gas constant (8.314 J mol^–1^ K^–1^), *H*_d_ is the energy of deactivation and Δ*S* is an entropy term (Hikosaka et al. 2006).

CO_2_ is transferred from air to the carboxylation site due to diffusion. There are three important limiting steps for CO_2_ diffusion: boundary layer, stomata, and mesophyll. Using Fick’s law, CO_2_ diffusion is expressed as follows:

13 $$A = g_{{\text{b}}} \left( {C_{a} - C_{{\text{f}}} } \right) = g_{{\text{s}}} \left( {C_{{\text{f}}} - C_{{\text{i}}} } \right) = g_{{\text{m}}} \left( {C_{{\text{i}}} - C_{{\text{c}}} } \right)$$

where *g*_b_, *g*_s_, and *g*_m_ are conductance for CO_2_ diffusion at boundary layer, stomata, and mesophyll, respectively, and *C*_a_, *C*_f_, and *C*_i_ are CO_2_ partial pressure at air, leaf surface, and intercellular space. Under normal conditions, boundary layer conductance is much greater than stomatal conductance so that it is often ignored or combined with stomatal conductance as leaf conductance.

Stomatal conductance changes depending on environmental conditions and is thus an important regulator for CO_2_ diffusion. Thus far, various models have been developed (see Buckley 2005; Buckley and Mott 2013; Damour et al. 2010 for review). Despite significant advances in molecular biology for environmental response of stomata, our understanding is still insufficient to make a mechanistic model for stomatal conductance. Instead, previous models have used empirical relationships, semi-mechanistic relationships, or optimality theories. Here, we introduce three simple models:

14 $$g_{s} = g_{0} + g_{1} \frac{{Ah_{{\text{r}}} }}{{C_{{\text{f}}} }}$$

15 $$g_{s} = g_{0} + g_{1} \frac{A}{{\left( {C_{{\text{f}}} - {\Gamma }} \right)\left( {1 + D/D_{0} } \right)}}$$

16 $$g_{s} = g_{0} + \left( {1 + \frac{{g_{1} }}{\sqrt D }} \right)\frac{A}{{C_{{\text{a}}} }}$$

where *g*_0_ and *g*_1_ are fitted parameters, *h*_r_ is relative humidity at the leaf surface, Γ is the CO_2_ compensation point of photosynthesis in the presence of respiration in the light, *D* is the leaf-to-air vapor pressure deficit (VPD), and *D*_0_ is a fitted parameter. Equations 14, 15, and 16 were proposed by Ball et al. (1987), Leuning (1995), and Medlyn et al. (2011), respectively. All these models predict that stomatal conductance is higher in lower atmospheric CO_2_ concentration, higher humidity, and the condition when the CO_2_ assimilation rate can be higher.

Mesophyll conductance has a similar importance to stomatal conductance for the CO_2_ assimilation rate; it has been shown that the difference in the CO_2_ partial pressure between the intercellular space and stroma is similar to that between air and intercellular space. Across species, mesophyll conductance is nearly proportional to stomatal conductance (Flexas et al. 2012; Loreto et al. 1992). It has been shown that mesophyll conductance changes in response to environmental changes; for example, it decreases with water stress (Flexas et al. 2013) and with elevated CO_2_ (Tazoe et al. 2011). However, the number of studies on environmental responses of mesophyll conductance is limited due partly to the difficulty of its measurement in the field. There seems to be no model to describe environmental dependence of mesophyll conductance.

## Remote sensing of photosynthesis-related parameters

Vegetation indices such as NDVI (Rouse et al. 1974) and enhanced vegetation index (EVI; Huete et al. 2002) mainly use reflectance at red (R) and near infra-red (NIR). For example, NDVI is calculated as follows:

17 $${\text{NDVI}} = \frac{{\rho_{{{\text{NIR}}}} - \rho_{{\text{R}}} }}{{\rho_{{{\text{NIR}}}} + \rho_{{\text{R}}} }}$$

where ρ_NIR_ and ρ_R_ are the reflectance at NIR and R, respectively. Because Chl preferentially absorbs R but not NIR, NDVI is higher when the Chl content of the vegetation is high. However, NDVI is not necessarily linearly related with the Chl content. Therefore, other indices have been developed to improve quantitative accuracy (for review, see Pontius et al. 2020).

Recently, Li et al. (2019) proposed a new index to determine the leaf Chl content remotely, MDatt, which is given as follows:

18 $${\text{MDatt}} = \frac{{\rho_{720} - \rho_{761} }}{{\rho_{720} - \rho_{672} }}$$

where ρ_X_ is the reflectance at X nm. This index is linearly correlated with the leaf Chl content and the correlation is not influenced by the observation angle, even when specular reflection occurs.

Chl fluorescence can be detected with several techniques. The PAM system cannot be used for remote sensing study because it requires a short distance between the leaves and system to apply artificial saturation flash and accurate pulse-synchronized and modulated fluorimetric techniques. Instead, the laser-induced Chl fluorescence transients (LIFT) method enables active Chl fluorescence measurement up to a distance of 50 m (Kolber et al. 2005; Pieruschka et al. 2014). LIFT uses low-intensity pulses to measure the fluorescence transient, which is interpolated to the maximum fluorescence level.

**Fig. 3:**
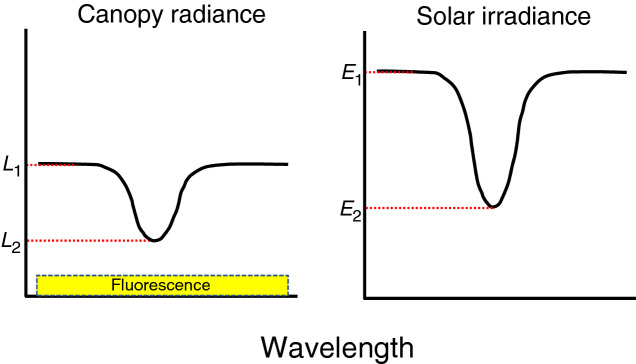
The Fraunhofer line depth principle. Solar irradiance in the line is depressed (*E*_2_), but chlorophyll fluorescence is emitted even in the line. Consequently, the ratio of *L*_1_ to *L*_2_ is different from the ratio of *E*_1_ to *E*_2_ due to addition of chlorophyll fluorescence

The Fraunhofer line depth principle enables detection of Chl fluorescence in a passive way (Plascyk 1975). The Fraunhofer line is the dark line occurring in specific wavelengths due to light absorption by molecules in the sun or earth atmosphere. In the Fraunhofer line, solar irradiance is weakened but fluorescence is emitted irrespective of the line (Fig. 3), because the wavelength of fluorescence is longer than that of inducing light (Fig. 1). The ratio of irradiance from the line to that from outside of the line is different between the radiation from the sun and that from the vegetation because the latter includes fluorescence. The intensity of solar-induced Chl fluorescence (SIF) can thus be calculated as follows:

19 $${\text{SIF}} = L_{2} - \frac{{L_{1} - L_{2} }}{{E_{1} - E_{2} }}E_{2}$$

where *E* and *L* indicate solar radiation and radiance from the canopy, respectively, and the subscripts ‘1’ and ‘2’ denote the reference and bottom of the absorption band, respectively (Fig. 3). Note that the value obtained by Eq. 19 is the absolute value of fluorescence (not the quantum yield). Equation 19 implicitly assumes that the ‘true’ reflectance at the reference and the bottom wavelength are identical to each other. However, this is not necessarily true in many cases. To overcome this problem, other methods have been developed such as 3FLD (Maier et al. 2003), iFLD (Alonso et al. 2008), and the spectrum fitting method (Meroni and Colombo 2006). These methods are reviewed by Meroni et al. (2009).

Photochemical reflectance index (PRI) is another index that can reflect from the biochemical state of the photosynthetic apparatus. Gamon and coworkers found that the reflectance around 530 nm was highly associated with the epoxidation state of the xanthophyll cycle (Gamon et al. 1990, 1992, 1993). The PRI is calculated as follows (Gamon et al. 1997; Peñuelas et al. 1995):

20 $${\text{PRI}} = \frac{{\rho_{531} - \rho_{570} }}{{\rho_{531} + \rho_{570} }}$$

**Fig. 4:**
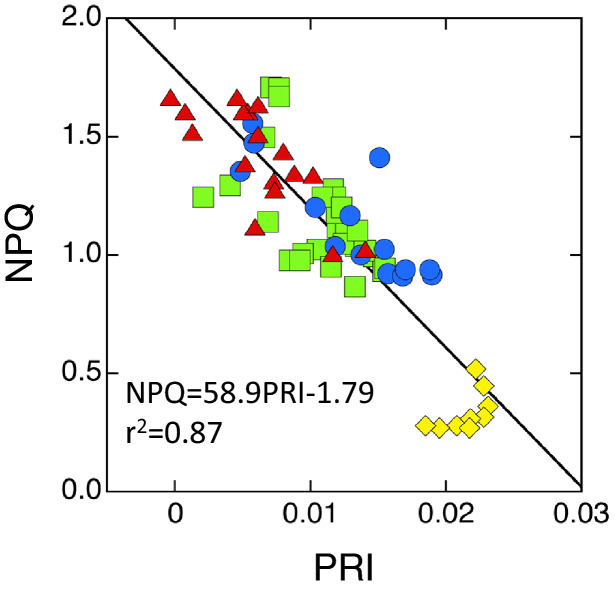
The relationship between non-photochemical quenching (NPQ) and photochemical reflectance index (PRI). Blue circles, red triangles, green squares, and yellow diamonds were obtained in different air CO_2_ concentrations, different water status (time after petiole cutting), different leaf temperatures, and low irradiance, respectively. The line is a linear regression. Redrawn from Hikosaka and Noda (2019) with modifications

PRI was shown to be correlated with NPQ (Evain et al. 2004; Hikosaka and Noda 2019; Rahimzadeh-Bajgiran et al. 2012; Fig. 4). It should be noted that PRI does not necessarily reflect the whole mechanism of NPQ because it changes only with the de-epoxidation state, but not with PsbS-related quenching. Kohzuma and Hikosaka (2018) showed that PRI was correlated with NPQ in the wild type and a mutant that lacks the PsbS protein (*npq4*), but not in a mutant that cannot convert violaxanthin to zeaxanthin due to inhibited activity of violaxanthin de-epoxidase (*npq1*). However, because both de-epoxidation of violaxanthin and PsbS protonation are induced by a decrease in lumen pH (Goss and Lepetit 2015), their environmental responses may be similar to each other and PRI can be used for assessment of NPQ in most plants.

## Environmental dependence of CO_2_ assimilation rate, chlorophyll fluorescence and PRI

### Leaf scale experiments

**Fig. 5:**
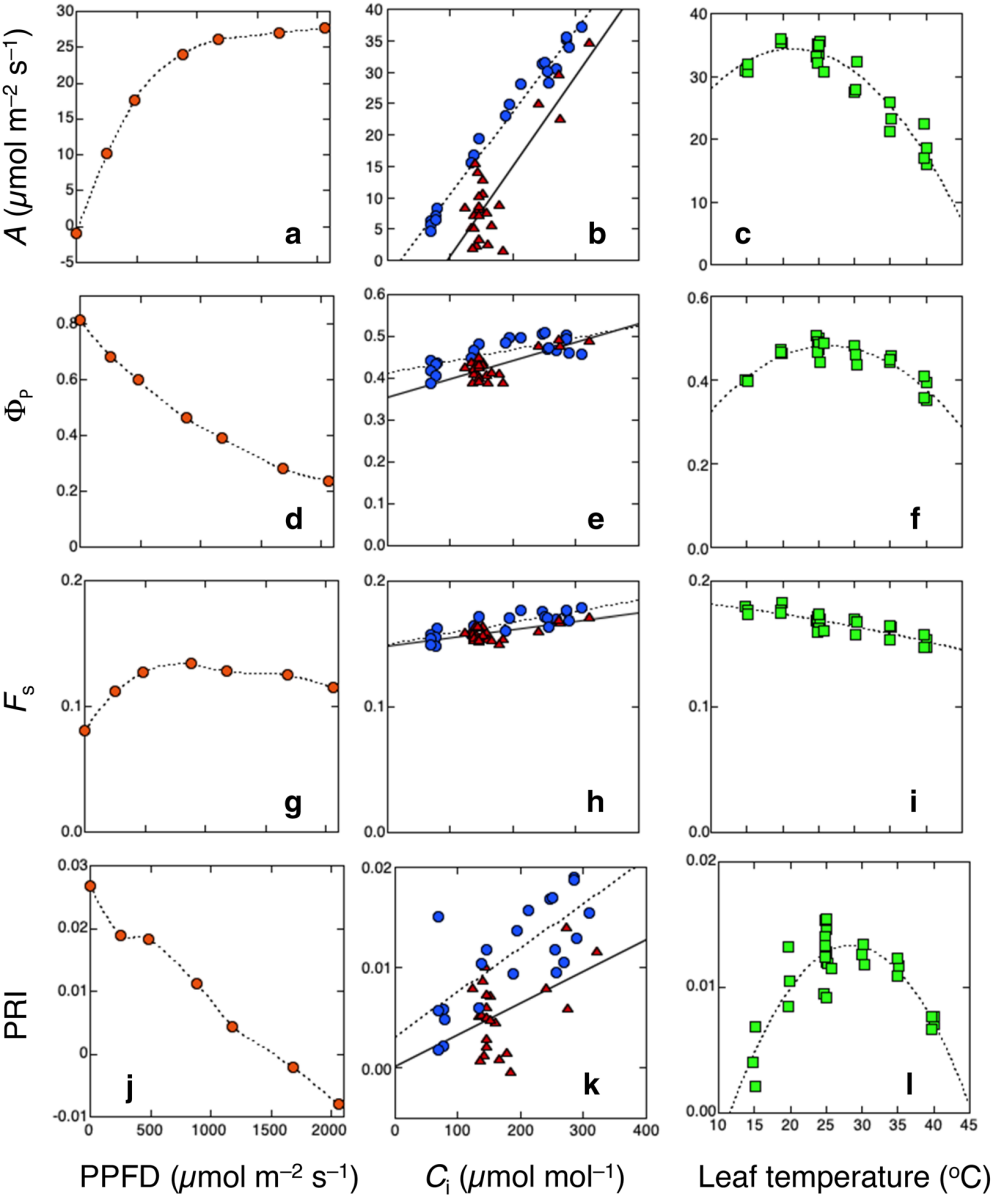
The CO_2_ assimilation rate (*A*; **a**–**c**), the quantum yield of photochemistry in the light (Φ_P_; **d**–**f**), the steady-state fluorescence level (*F*_s_; **g**–**i**), and photochemical reflectance index (PRI; **j**–**l**) as a function of light (**a**, **d**, **g**, **j**), intercellular CO_2_ concentration (*C*_i_; **b**, **e**, **h**, **k**), and leaf temperature (c; **f**, **i**, **l**) in *Chenopodium album* leaves. In **b**, **e**, **h**, and **k**, blue circles and red triangles were obtained in different air CO_2_ concentrations and different water status (time after petiole cutting), respectively. Basic measurement condition was 1200 µmol m^−2^ s^−1^ PPFD, 25 °C leaf temperature, and 400 µmol CO_2_ mol^−1^. The lines are interpolation, linear or quadratic regression. **a**, **d**, **g**, and **j** are redrawn from Tsujimoto and Hikosaka (2021) and others are from Hikosaka and Noda (2019) with modifications

Figure 5 shows results of a simultaneous measurement of CO_2_ assimilation rates, Chl fluorescence, and PRI in *Chenopodium album* leaves under various measurement conditions (Hikosaka and Noda 2019; Tsujimoto and Hikosaka 2021). As has been well known, CO_2_ assimilation rates were decreased by decreasing irradiance (Fig. 5a), decreasing atmospheric CO_2_ concentration (Fig. 5b), water stress (Fig. 5b), and extremely lower and higher temperatures (Fig. 5c). Φ_P_, photochemistry per absorbed PPFD, was decreased by increasing irradiance (Fig. 5d) because the electron transport rate was saturated at higher irradiance (note that CO_2_ assimilation per absorbed PPFD also decreases with increasing PPFD). Φ_P_ was also decreased by decreasing CO_2_ concentration (Fig. 5e), water stress (Fig. 5e), and extremely lower and higher temperatures (Fig. 5f).

Environmental responses of the steady-state Chl fluorescence signal, *F*_s_, which is proportional to Φ_F_, were similar to those of Φ_P_ in CO_2_ and water stress responses; *F*_s_ was decreased by decreasing CO_2_ concentration and water stress (Fig. 5h). On the other hand, the light and temperature responses of *F*_s_ slightly differed from those of Φ_P_. *F*_s_ showed a parabolic curve against irradiance; it was increased by increasing PPFD when PPFD was low but was slightly decreased when PPFD was high (Fig. 5g). *F*_s_ was monotonically decreased by increasing temperature (Fig. 5i). Therefore, environmental responses of Φ_F_ are not necessarily the same as those of Φ_P_. In contrast, environmental responses of PRI were relatively similar to those of Φ_P_; PRI was decreased by increasing PPFD (Fig. 5j), decreasing CO_2_, water stress (Fig. 5k), and extremely low and high temperatures (Fig. 5l).

These environmental responses of *F*_s_ can be interpreted as follows. With increasing light intensity, the fraction of energy that can be consumed by photochemistry decreases because of the limitation by downstream processes in CO_2_ fixation and photorespiration. When PPFD increases from low to intermediate, NPQ change is relatively small and thus the energy that cannot be consumed by photochemistry is allocated to fluorescence, leading to an increase in fluorescence. When PPFD increases from intermediate to high and photosynthesis is saturated, NPQ becomes active so that energy allocated to photochemistry and fluorescence is decreased. When the CO_2_ concentration or water availability is decreased, or when the leaf temperature is high, NPQ becomes active so that the energy allocated to photochemistry and fluorescence is decreased (Flexas et al. 2002). When temperature is low, photochemistry is suppressed and NPQ is activated. However, the activation of NPQ is not sufficient and energy allocated to fluorescence is slightly increased. These interpretations are consistent with a meta-analysis of field observations by Ač et al. (2015); whereas the steady-state Chl fluorescence decreased in water or heat stress, it increased under chilling stress.

**Fig. 6:**
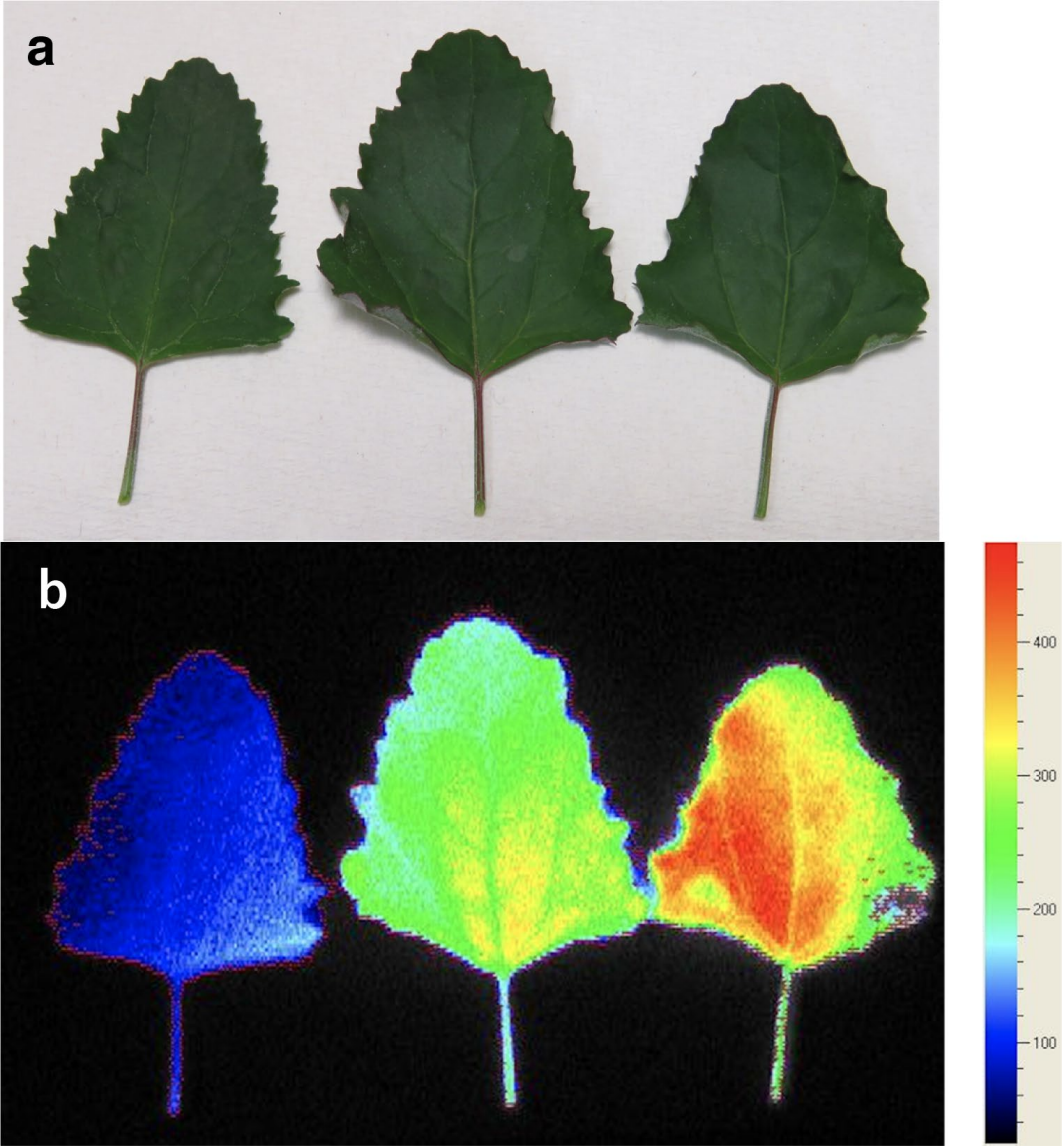
Images of artificially-photoinhibited and control leaves of *Chenopodium album*. Upper image was taken by a normal camera. Lower image represents the chlorophyll fluorescence in the dark (*F*_o_) taken by a two-dimensional chlorophyll fluorescence imaging system (FluorCam, PSI). All leaves were treated with lincomycin, which inhibits repair of damaged PSII. Left, middle, and right leaves were exposed to strong light 0, 3, and 9 h, respectively. Red and blue represent higher and lower *F*_o_ values, respectively

**Fig. 7:**
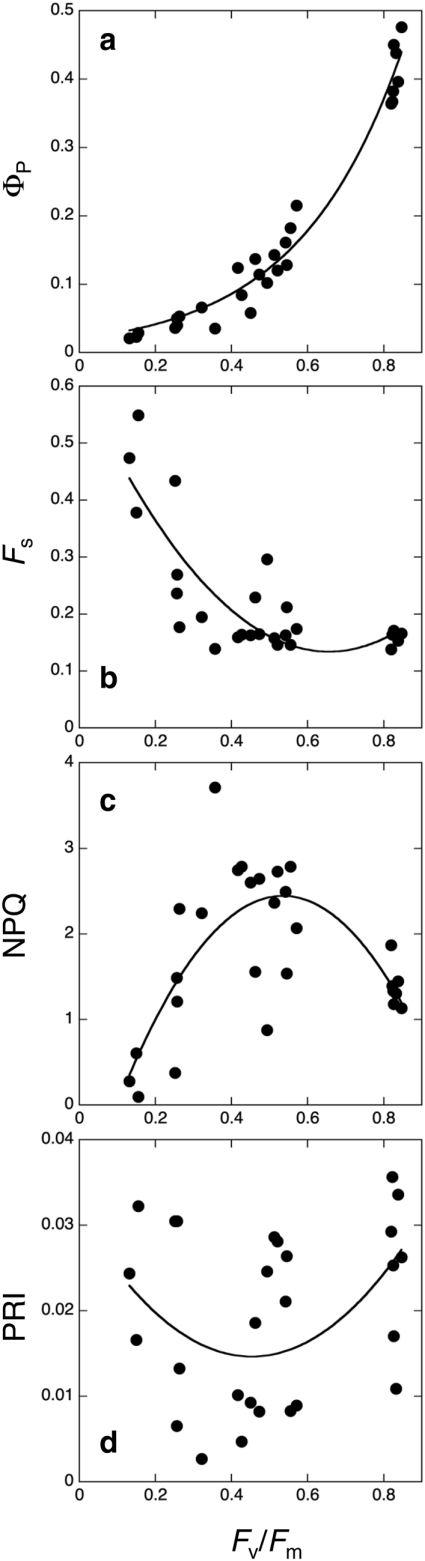
The quantum yield of photochemistry in the light (Φ_P_; **a**), the steady-state fluorescence level (*F*_s_; **b**), non-photochemical quenching (NPQ; **c**), and photochemical reflectance index (PRI; **d**) as a function of *F*_v_/*F*_m_, which is a measure of the degree of photoinhibition, in artificially photoinhibited leaves of *Chenopodium album*. NPQ of photoinhibited leaves was calculated using *F*_m_ value of non-photoinhibited leaves. The lines are exponential or quadratic regressions, which were selected from various curves based on Akaike Information Criterion. Redrawn from Hikosaka (2021) with modifications

As mentioned in the first section, when plants are exposed to stress, the rate of photodamaged PSII often becomes higher than its recovery rate, leading to photoinhibition. Because photochemistry does not occur in damaged PSII, energy partitioning in PSII is very different between photoinhibited and healthy leaves. It is known that fluorescence yield in the dark (*F*_o_) is often very high in photoinhibited leaves of some species (Demmig et al. 1987; Hong and Xu 1999; Hikosaka 2021; Fig. 6). Hikosaka (2021) investigated energy partitioning in artificially photoinhibited leaves of *C. album*. *F*_v_/*F*_m_ was used as a measure of photoinhibition. Φ_P_ decreased with decreasing *F*_v_/*F*_m_ (Fig. 7a). *F*_s_ did not change when *F*_v_/*F*_m_ was 0.8–0.6, but remarkably increased with decreasing *F*_v_/*F*_m_ from 0.6 (Fig. 7b). NPQ increased when *F*_v_/*F*_m_ changed from 0.8 to 0.6, but decreased when *F*_v_/*F*_m_ was lower than 0.6 (Fig. 7c). Inversely, PRI decreased with decreasing *F*_v_/*F*_m_ from 0.8 to 0.6, and increased when *F*_v_/*F*_m_ was lower than 0.6 (Fig. 7d). In *C. album* leaves, NPQ processes safely dissipate the excess energy when the photoinhibition is not severe (*F*_v_/*F*_m_ > 0.6), leading to a relatively stable *F*_s_. However, when the photoinhibition is severer (*F*_v_/*F*_m_ < 0.6), NPQ is decreased, leading to an increase in *F*_s_. These results suggest that the relationship among photochemistry, NPQ, and fluorescence is not simple. When NPQ can dissipate excess energy sufficiently, energy allocation to NPQ is increased under stress conditions to protect PSII and energy allocation to photochemistry and fluorescence is reduced (low CO_2_ concentrations, water deficiency, and high temperature). In this situation, Φ_P_ and Φ_F_ would be positively related to each other along the gradient of stress conditions (Flexas et al. 2002). On the other hand, when the capacity of NPQ is limited, the energy that cannot be used in NPQ nor photochemistry will be allocated to other processes including fluorescence (low temperature and photoinhibition). In this situation, the relationship between Φ_P_ and Φ_F_ would be negative (Hikosaka 2021). Therefore, the slope of the relationship between Φ_P_ and Φ_F_ changes depending on environmental conditions. In addition, it should be noted that increases of *F*_o_ in photoinhibited leaves are not observed in some species (Hong and Xu 1999), suggesting interspecific variations in light energy partitioning in photoinhibited leaves.

### Field observations

In remote sensing studies, GPP has been obtained from vegetation indices such as NDVI and EVI using the light use efficiency (LUE) model proposed by Monteith (1972).

21 $${\text{GPP}} = {\text{LUE}} f_{{{\text{APAR}}}} {\text{PAR}}$$

where PAR is photosynthetic active radiation above the canopy, *f*_APAR_ is the fraction of PAR absorbed by the canopy, and LUE is GPP divided by absorbed PAR (Field et al. 1995). *f*_APAR_ is obtained as a function of vegetation indices (Sims et al. 2006; Xiao et al. 2004). However, LUE is calculated from empirical functions of the maximal LUE and suppression by environmental variables, which could not be deduced from vegetation indices.

PRI is expected to be useful to assess LUE by remote sensing. Many studies have reported that PRI is positively related to LUE at a leaf (e.g., Gamon et al. 1997; Nakaji et al. 2006) and canopy scale (e.g., Garbulsky et al. 2008; Kováč et al. 2020). Garbulsky et al. (2011) performed a meta-analysis for the relationship between LUE and PRI. From leaf to canopy and ecosystem scales, LUE was significantly correlated with PRI. The LUE-PRI relationship was generally exponential, i.e., the increase of LUE with PRI was greater at higher PRI values. The relationship was slightly different among vegetation types. For example, when compared at the same PRI, canopy-level LUE was higher in conifers than in herbaceous plants. In each vegetation type, PRI explained more than 40% of the variations in GPP. However, the correlation between GPP and the PRI is often non-significant (e.g., Drolet et al. 2008; Nakaji et al. 2014), because PRI is influenced by factors other than xanthophylls. This is discussed in the next section.

SIF has been reported to be positively related with GPP both in site (Li et al. 2020; Magney et al. 2019; Miao et al. 2018; Yang et al. 2015) and satellite observations (Frankenberg et al. 2011; Guanter et al. 2014; Parazoo et al. 2014; Sun et al. 2017; Verma et al. 2017; Yang et al. 2017; Zhang et al. 2014). Why is the relationship between GPP and SIF positive, though absorbed energy is competitively allocated between photochemistry and fluorescence? Several mechanisms may be involved in this relationship. First, both GPP and SIF are higher when the amount of Chl in the vegetation (i.e., the leaf area index) is higher. Second, both GPP and SIF are higher when irradiance above the stand is higher. Third, a positive relationship between Φ_P_ and Φ_F_ is caused by changes in NPQ; because an increase in NPQ decreases both Φ_P_ and Φ_F_, Φ_P_ and Φ_F_ are higher and lower in healthy and stressed leaves, respectively (Flexas et al. 2002). The slope for the GPP–SIF relationship often differs depending on the species composition of the canopy. For example, Zhang et al. (2020) found that the relationships between observed canopy-leaving SIF and ecosystem GPP varied significantly among C_3_ grasslands, C_4_ corn fields, and temperate deciduous forests.

## Theoretical linkages between remote sensing parameters and the CO_2_ assimilation rate

### Estimation of CO_2_ exchange rates from electron transport rate and stomatal coefficient

Although the limiting step of CO_2_ assimilation changes depending on environmental conditions as shown in Eqs. 6–8, the relationship between CO_2_ assimilation rate (*A*) and the electron transport rate (*J*) can be described in Eq. 7, because electron transport rate is down-regulated when the CO_2_ assimilation rate is limited by other processes. *J* can be assessed from Chl fluorescence and/or PRI as discussed below.

The rate of RuBP regeneration is tightly related to the rate of linear electron transport, but the fraction of RuBP used for carboxylation or oxygenation changes depending on CO_2_ concentration in the chloroplast (Farquhar et al. 1980). As mentioned above, CO_2_ concentration in the chloroplast depends on stomatal and mesophyll conductance, both of which are hardly assessed in remote sensing. Stomatal conductance can be estimated if environmental variables and stomatal coefficients are available as shown in Eqs. 14–16. Stomatal coefficients are variable depending on species and climates. Based on a meta-analysis, Lin et al. (2015) proposed a model to predict *g*_1_ in Eq. 16 across plant functional types and across biomes. It should be noted that Eqs. 14–16 assume that water stress influences photosynthesis only through a decrease in humidity. If the water stress is caused by low soil water availability rather than by low humidity, Eqs. 14–16 cannot correctly predict a decrease in stomatal conductance because these equations do not consider soil conditions. Some stomatal models incorporate water potential within the leaf, but evaluation of leaf water potential is not necessarily possible in remote sensing. However, information related to plant water status such as leaf water content can be detected by satellite observation (Hunt et al. 2013). Bayat et al. (2019) proposed a modification of the radiative transfer model SCOPE to predict water stress effects on GPP. They showed that the predictability of GPP under water stress is improved by incorporating vapor pressure, suppression of *V*_cmax_ by a soil moisture dependent stress factor, and the soil surface resistance.

Mesophyll conductance is often assumed to be infinite, which allows calculation of Rubisco kinetic parameter values (i.e. *V*_max_ and *K*_m_ for carboxylation and oxygenation). In this calculation, variations in *V*_cmax_ (termed as ‘apparent *V*_cmax_’) involve changes in the mesophyll conductance. This method can allow to simulate environmental dependence of CO_2_ assimilation rates in most cases. However, it has been suggested that lack of mesophyll conductance results in strongly biased estimates of net assimilation due to too strong CO_2_ gradients under water stress (Niinemets et al. 2009; Niinemets and Keenan 2014). Because apparent *V*_cmax_ potentially involves mesophyll conductance*,* the assumption of suppression in *V*_cmax_ by water stress proposed by Bayat et al. (2019) may be reasonable to practically predict GPP under water stress.

### Estimation of electron transport rate from the quantum yield of PSII photochemistry

As the linear electron transport rate in PSII is a product of Φ_P_ and the absorbed PPFin PSII, *J* is expressed as follows:

22 $$J = \alpha \beta \eta I{\Phi }_{{\text{P}}}$$

where *α*, *β, η* and *I* are the leaf absorptance of PPFD, the fraction of light absorbed by PSII Chl, the fraction of reducing power used for the Calvin-Benson cycle and photorespiration, and incident PPFD, respectively. In many studies, *α* is assumed to be 0.84 (e.g., Schreiber et al. 1995), but *α* changes depending on leaf Chl content (Gabrielsen 1948). *α* is saturated if leaf Chl content is high enough (e.g. > 0.4 g Chl m^−2^), but decreases when leaf Chl content is very low (e.g., senesced or nutrient deficient leaves). Structure of leaf surface such as pubescence may also influence *α* (e.g., Ehleringer et al. 1976). *β* is generally assumed to be 0.5, but it may change depending on growth conditions. For example, when leaves are grown under enriched far-red light conditions, the fraction of PSII Chl increases (Wientjes et al. 2017).

*η* has been ignored in most previous studies; i.e., it was implicitly assumed to be 1. However, reducing power produced by the thylakoid reaction is used for processes other than CO_2_ fixation and photorespiration. For example, plants use this reducing power for various metabolisms including nitrogen and sulfur assimilation (Hanke and Mulo 2013). In the water-water cycle, reducing power is consumed to dissipate excess energy; an electron is transferred from PSI to O_2_ and the produced reactive oxygen species are safely removed as water using reduction power (Asada 1999). Although the water-water cycle is an important sink for the dissipation of excess excitation energy in cyanobacteria (Badger et al. 2000) and diatoms (Waring et al. 2010), O_2_ exchange measurements using mass spectrometry suggested that it is a minor sink in higher plants even when photosynthesis and photorespiration are suppressed (Driever and Baker 2011). However, several studies have observed uncoupling of linear electron transport and energy consumed by CO_2_ assimilation plus photorespiration under certain conditions (e.g., Driever and Baker 2011; Makino et al. 2002; Miyake and Yokota 2000), suggesting that alternative electron flows may influence *η*.

### Estimation of quantum yield of PSII photochemistry from chlorophyll fluorescence yield

**Fig. 8:**
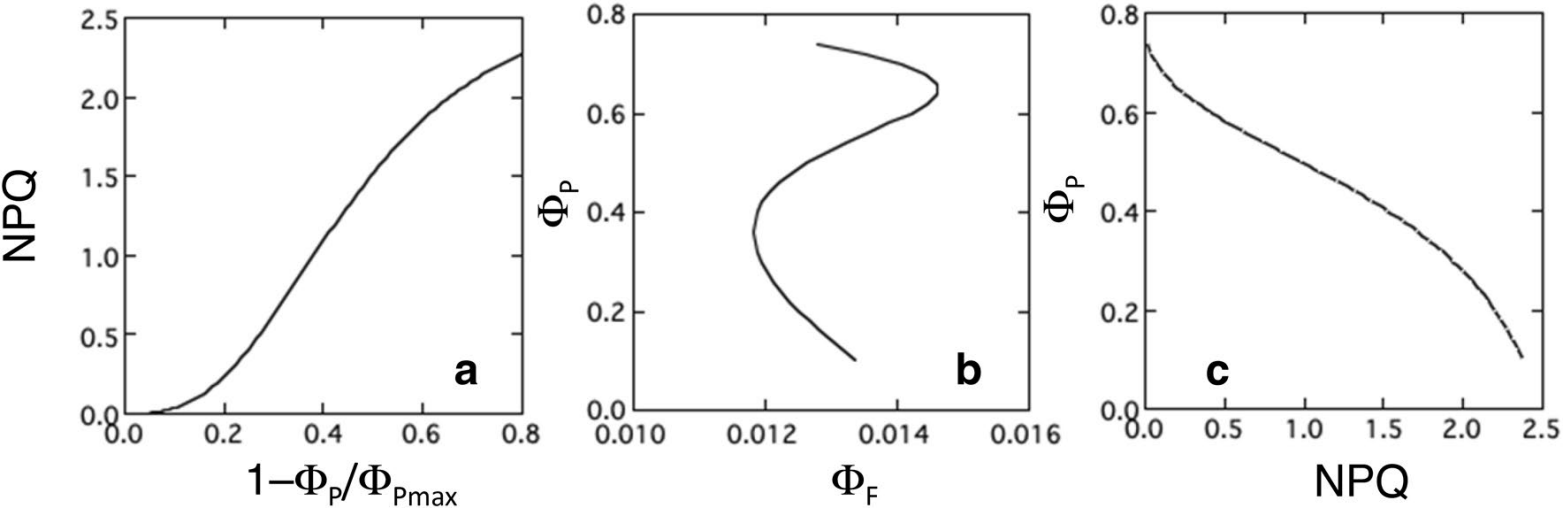
The relationships between energy allocation among photochemistry, fluorescence, and non-photochemical quenching (NPQ). NPQ is expressed as a function of 1–Φ_P_/Φ_Pmax_ (**a**). Φ_P_ is expressed as a function of Φ_F_ (**b**) and NPQ (**c**). Lines are drawn based on Eq. 23. See text for detail

While Φ_P_ can be easily determined in the PAM system, it cannot be obtained directly in remote sensing. The Fraunhofer line depth method detects only SIF, i.e., steady-state fluorescence intensity in the light. Φ_F_ can be obtained from the SIF divided by the absorbed PPFD, but energy allocation to other processes cannot be known. Since *K*_N_ and *K*_P_ are considered to vary depending on environmental variables, some assumption is necessary to derive Φ_P_ from SIF. van der Tol et al. (2014) proposed an empirical model to describe the relationship between photochemistry and NPQ.

23 $$K_{{\text{N}}} = \frac{{\left( {1 + \gamma } \right)x^{\delta } }}{{\gamma + x^{\delta } }}K_{{{\text{Nmax}}}}$$

where γ and δ are fitting parameters, *K*_Nmax_ is the maximum *K*_N_ and *x* is the relative light saturation given as follows.

24 $$x = 1 - \frac{{{\Phi }_{{\text{P}}} }}{{{\Phi }_{{{\text{Pmax}}}} }}$$

where Φ_Pmax_ is the maximum Φ_P_, which may be assumed to be *F*_v_/*F*_m_. van der Tol et al. (2014) adopted 0.05, 0.95, 2.48, 2.83, and 0.114 for *K*_F_, *K*_D_, *K*_Nmax_, γ and δ, respectively. Figure 8a shows NPQ (NPQ = *K*_N_ as *K*_D_ + *K*_F_ = 1) as a function of 1-Φ_P_/Φ_Pmax_.

Equation 23 assumes that NPQ is greater when the energy becomes more excessive for photochemistry. van der Tol et al. (2014) tested the validity of Eq. 23 using published datasets. The relationship between *K*_N_ and *x* was similar irrespective of light intensity and CO_2_ concentration at the measurement, photosynthesis type (i.e., C_3_ or C_4_), and fertilization levels, suggesting that this relationship can be applied to various cases. However, they also reported that the relationships in drought-adapted plants were slightly different from those in other plants. Recently, Hikosaka (2021) demonstrated that this relationship is not held in severely photoinhibited leaves as discussed above. Therefore, this equation needs careful application.

From Eq. 1, Φ_F_ can be given as follows:

25 $${\Phi }_{{\text{F}}} = \frac{{{K}_{{\text{F}}} }}{{{K}_{{\text{F}}} + {K}_{{\text{D}}} + {K}_{{\text{N}}} + {K}_{{\text{P}}} }} = \frac{{{K}_{{\text{F}}} }}{{{K}_{{\text{F}}} + {K}_{{\text{D}}} + {K}_{{\text{N}}} }}\frac{{{K}_{{\text{F}}} + {K}_{{\text{D}}} + {K}_{{\text{N}}} }}{{{K}_{{\text{F}}} + {K}_{{\text{D}}} + {K}_{{\text{N}}} + {K}_{{\text{P}}} }} = \frac{{{K}_{{\text{F}}} }}{{{K}_{{\text{F}}} + {K}_{{\text{D}}} + {K}_{{\text{N}}} }}\left( {1 - {\Phi }_{{\text{P}}} } \right)$$

Substituting Eqs. 23 to 25, Φ_F_ can be expressed as the function of Φ_P_. Figure 8b shows Φ_P_ as a function of Φ_F_ using values shown in van der Tol et al. (2014). The relationship is not simple; it is negative when Φ_P_ is high and low and positive when Φ_P_ is intermediate. In some regions, there are three possible values of Φ_P_ to satisfy Eq. 25 at a given Φ_F_. Therefore, it is not easy to estimate Φ_P_ solely from Φ_F_. Because Φ_F_ is low at low and high light and highest at intermediate light (Fig. 5c), light availability needs to be considered together to relate Φ_F_ to Φ_P_.

Practically, correct estimation of Φ_F_ is not easy work. Because fluorescence is emitted in all directions, an integration sphere such as FluoWat leaf clip (Amoros-Lopez et al. 2008) may be necessary for correct assessment, but not realistic in remote sensing. In the PAM system, *F*_s_ is expected to be proportional to Φ_F_, but its value changes with the distance between the leaf and fiber and with the intensity of the measuring beam. The FLD methods can determine the absolute value of emitted energy. However, the SIF value obtained by the FLD methods reflects only in the Fraunhofer line and the spectrum of Chl fluorescence needs to be considered to calculate Φ_F_. Fluorescence from PSI Chl may influence SIF. Several studies have compared SIF yield (SIF divided by the absorbed light) with *F*_s_ (e.g., Cendrero-Mateo et al. 2016; Helm et al. 2020). These studies showed that SIF yield is significantly correlated with *F*_s_, but the relationship is not necessarily proportional. These results suggest that SIF is useful to estimate Φ_F_, but should be treated carefully when the value is used quantitatively.

### Estimation of quantum yield of PSII photochemistry from PRI

It has been shown that NPQ is negatively correlated with PRI (Garbulsky et al. 2011; Fig. 4). The regression is linear in many cases (Hikosaka and Noda 2019; Rahimzadeh-Bajgiran et al. 2012; Fig. 3), but curvilinear in some reports (Evain et al. 2004). Here, linear relationship is applied.

26 $${\text{NPQ }} = c + d{\text{PRI}}$$

where *c* and *d* are the intercept and slope, respectively. Transformation of Eq. 23 enables to obtain Φ_P_ from NPQ (NPQ = *K*_N_).

27 $${\Phi }_{{\text{P}}} = {\Phi }_{{{\text{Pmax}}}} \left\{ {1 - \left[ {\frac{{\gamma {\text{NPQ}}}}{{K_{{{\text{Nmax}}}} \left( {1 + \gamma - \frac{{{\text{NPQ}}}}{{K_{{{\text{Nmax}}}} }}} \right)}}} \right]^{{\frac{1}{\delta }}} } \right\}$$

Φ_P_ gradually decreases with increasing NPQ (Fig. 8c). Substituting Eqs. 26 to 27, Φ_P_ can be obtained from PRI.

One of the problems for the use of PRI to assess NPQ is that the relationship between NPQ and PRI varies among leaves. Its slope (*d* in Eq. 26) seems common among leaves, but the intercept (*c*) has large variations. This is because PRI is influenced not only by the de-epoxidation state of the xanthophyll cycle but also by composition of other pigment concentrations, such as Chls and carotenoids (Gamon and Berry 2012; Gitelson 2020; Gitelson et al. 2017; Nakaji et al. 2006). To overcome this problem, many studies have used the difference between the PRI value in the light and that in the dark (ΔPRI; Gamon and Surfus 1999). PRI in the dark, termed PRI_0_, is defined as the PRI value when NPQ is zero. Compared with PRI, ΔPRI is a better predictor for NPQ and Φ_P_ across different leaves (e.g., Gamon et al. 1997; Hmimina et al. 2014; Kováč et al. 2020). However, determination of PRI in the dark is not necessarily easy in field observations. Previous field studies have determined PRI_0_ in a period around 9:00 AM, when solar angle is low (Liu et al. 2013; Magney et al. 2016), but solar irradiance may be too strong to obtain correct PRI_0_ (Kováč et al. 2020). Observation in early morning is not possible from satellite platforms. Several studies have tried to predict PRI_0_ from leaf reflectance spectra. Rahimzadeh-Bajgiran et al. (2012) showed that a variation in the intercept of the NPQ–PRI relationship in *Solanum melongena* leaves can be calibrated using the red-edge normalized difference vegetation index (mNDVI_705_; Gitelson and Merzlyak 1994). Merlier et al. (2017) also showed that PRI_0_ was correlated with the modified red-edge normalized difference vegetation index (mNDI_705_; Sims and Gamon 2002) for the leaves exposed to ozone and water deficit stresses. Recently, Tsujimoto and Hikosaka (2021) showed that another reflectance index, NDVI_green_ (Gitelson et al. 1996), is effective to estimate PRI_0_ of *C. album* leaves grown at different light and nutrient conditions. However, the generality of such corrections is still to be studied.

### Estimation of quantum yield of PSII photochemistry from Chl fluorescence and PRI

Quantum yield of photochemistry should be estimated from either of Chl fluorescence or PRI if Eq. 23 is always held between Φ_P_ and NPQ. However, this is not necessarily true, especially when the leaves are exposed to severe stress as discussed above. When the relationship between Φ_P_ and NPQ is not constant, Φ_P_ can be assessed if both NPQ and chlorophyll fluorescence are obtained simultaneously. Cheng et al. (2013) conducted a correlation analysis, which indicated that GPP is better predicted when both SIF and PRI were incorporated as independent variables than when either of them was considered alone. Hikosaka and Noda (2019) developed a model to predict CO_2_ assimilation rates from the steady-state Chl fluorescence and PRI. According to Atherton et al. (2016), Φ_P_ can be expressed as follows:

28 $${\Phi }_{{\text{P}}} = 1 - \frac{{{\Phi }_{{\text{F}}} \left( {1 + {\text{NPQ}}} \right)\left( {K_{{\text{F}}} + K_{{\text{D}}} } \right)}}{{K_{{\text{F}}} }}$$

**Fig. 9:**
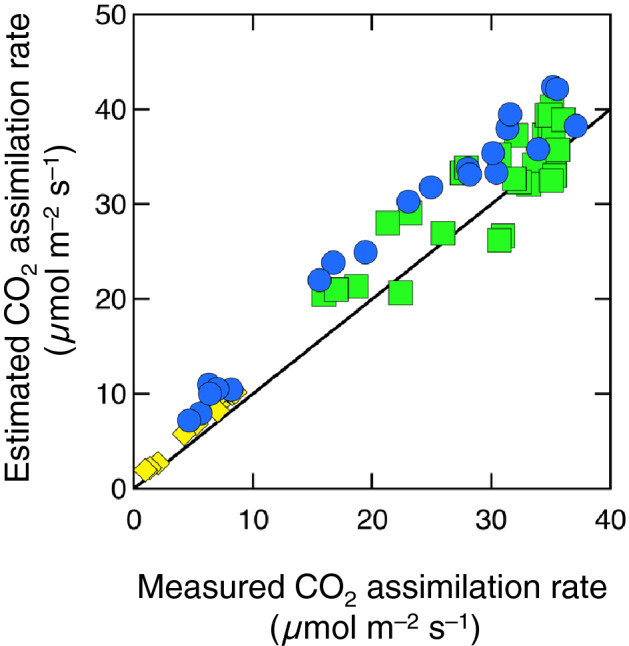
The relationship between the estimated and measured CO_2_ assimilation rate of *Chenopodium album* leaves exposed to various environments. Blue circles, green squares, and yellow diamonds were obtained in different air CO_2_ concentrations, different leaf temperatures, and low irradiance, respectively. The line is 1:1. Redrawn from Hikosaka and Noda (2019) with modifications

Hikosaka and Noda (2019) used the PAM system to obtain the steady-state chlorophyll fluorescence and modified Eq. 27 as follows:

29 $${\Phi }_{{\text{P}}} = 1 - b^{\prime}F_{{\text{s}}} \left( {1 + {\text{NPQ}}} \right)$$

where *F*_s_ is the steady-state fluorescence signal detected in the PAM system, which is proportional to Φ_P_, and *b'* = (*K*_F_ + *K*_D_)Φ_P_/(*K*_F_*F*_s_). NPQ was predicted from PRI (Eq. 26). Then, the CO_2_ assimilation rate was predicted using Eqs. 7, 14, and 22. The estimated CO_2_ assimilation rate was strongly related to the actual rate measured under various CO_2_, light, and temperature conditions (Fig. 9). A significantly positive relationship was also observed when the model was applied to photoinhibited leaves (Hikosaka 2021). Therefore, this model is applicable even when the relationship between Φ_P_ and NPQ varies.

## Conclusion

SIF and PRI are powerful tools to remotely assess gas exchange rates of plants. Since they reflect energy allocation within the photosystems, the quantum yield of photochemistry can be estimated from them. In many cases, energy allocation to NPQ can be assumed as a function of the quantum yield of photochemistry (Eq. 23), which enables to estimate the quantum yield of photochemistry from either SIF or PRI. However, this equation is not necessarily held in some cases such as low temperature and severe photoinhibition, suggesting that the quantum yield of photochemistry in such situations should be assessed carefully. When both SIF and PRI are used, photosynthesis may be estimated more accurately. Even when the quantum yield of photochemistry is correctly estimated, CO_2_ level in the chloroplasts requires incorporation for estimation of CO_2_ assimilation rate. Since it cannot be estimated by remote sensing directly, global analysis of stomatal conductance coefficient is important. Models linking Chl fluorescence and PRI with CO_2_ assimilation rates will contribute to understanding and future prediction of the global carbon cycle and climate change.
